# Measurements and scaling of X-ray total scattering from single crystals

**DOI:** 10.1107/S1600576726006187

**Published:** 2026-07-24

**Authors:** Semën Gorfman, Maksim Eremenko, Victor Krayzman, Alexei Bosak, Peter Y. Zavalij, Igor Levin

**Affiliations:** a Department of Materials Science and Engineering Tel Aviv University Tel Aviv Israel; b Materials Measurement Science Division National Institute of Standards and Technology (NIST) Gaithersburg MD20899 USA; c Spallation Neutron Source Oak Ridge National Laboratory Oak Ridge TN USA; d Theiss Research, La Jolla, CA, USA; e European Synchrotron Radiation Facility Grenoble France; f Department of Chemistry and Biochemistry University of Maryland College Park MD20742 USA; University of Tennessee, USA; Oak Ridge National Laboratory, USA

**Keywords:** diffuse scattering, total scattering, intensity scaling, structural refinement

## Abstract

This contribution presents a measurement protocol and a data-reduction workflow for obtaining single-crystal X-ray total-scattering datasets that capture both Bragg-peak and diffuse-scattering intensities on an absolute (electrons^2^ per atom) scale. It demonstrates that the intensity scale factor derived from crystallographic refinements using Bragg peaks is in reasonable agreement with the scale obtained by matching the scattering function, computed via spherical integration of the 3D total-scattering signal, to the theoretical coherent baseline. This baseline is calculated from the Debye–Waller factor. The latter scaling approach can be applied to diffuse scattering without including Bragg peaks. These results lay the groundwork for structural refinements using large atomic configurations while simultaneously fitting Bragg intensities and diffuse scattering from a single crystal. Moreover, with the convergence between the two scaling methods, the Bragg and diffuse components can be obtained from the same total-scattering dataset, as achieved in this work, or measured independently.

## Abstract 2

A measurement protocol and data reduction workflow are presented for obtaining single-crystal X-ray total-scattering datasets on an absolute scale, capturing both Bragg and diffuse intensities from the same dataset. The study demonstrates consistency between scale factors derived from Bragg refinements and from spherical integration of the total-scattering signal against a theoretical baseline, paving the way for structural refinements that simultaneously fit both contributions.

## 1. Introduction

X-ray or neutron total scattering performed on powder samples has become a common method for analyzing the local and nanoscale structure in crystalline materials. The total scattering includes Bragg peaks that represent the average periodic atomic arrangement, along with a smoothly varying diffuse background that contains information about locally correlated deviations from this average (Egami & Billinge, 2003 ▸). The Fourier transform of the total signal produces an atomic pair distribution function (PDF). Since interatomic correlations are often anisotropic, the resulting diffuse scattering is also anisotropic (Welberry, 2010 ▸). The orientational averaging inherent in powder measurements masks this anisotropy, limiting the amount of information that can be extracted from such data.

Recent results demonstrated that combining one-dimensional (1D) total-scattering data from powders with three-dimensional (3D) diffuse scattering from a single crystal enables recovery of occupational and displacement correlations that would be undetectable from powder data alone (Eremenko *et al.*, 2019 ▸; Krayzman *et al.*, 2022 ▸; Eremenko *et al.*, 2025 ▸). Such combined analysis, while powerful, requires both forms of the same material, assumes identical elemental composition and structure for both samples, and requires coordinating measurements across different facilities and instruments, each of which demands specialized expertise. Measurements under non-ambient conditions introduce complications, as variations in sample size and environment can affect controlled parameters. Therefore, if single crystals are available, it would be advantageous to be able to generate quantitative models of correlated atomic disorder across multiple length scales without requiring powder measurements.

One emerging approach toward this goal is the 3D ΔPDF method (Weber & Simonov, 2012 ▸), which exploits the Fourier transform of the diffuse intensity with Bragg peaks removed. A 3D ΔPDF exhibits positive and negative peaks at or around locations that correspond to distance vectors connecting different pairs of atoms in the average structure. These peaks reflect deviations from the average probability of finding a specific pair. Analyzing the signs, intensities, locations and shapes of these peaks allows determination of the correlations underlying diffuse scattering. While powerful, existing analysis methods for 3D ΔPDF do not produce fully atomistic structural models that would show explicitly how the local structure evolves into the average.

Another solution could involve measuring both Bragg peaks and diffuse scattering from a single crystal in the same dataset and using it to refine large atomic configurations, similar to the current use of powder total-scattering data with the reverse Monte Carlo (RMC) minimization method (Tucker *et al.*, 2007 ▸; Eremenko *et al.*, 2017 ▸; Zhang *et al.*, 2020 ▸). Such data, potentially augmented by direction-resolved X-ray absorption fine structure (Krayzman *et al.*, 2009 ▸), can be expected to yield highly detailed structural models. The key experimental hurdle is achieving an adequate signal-to-noise ratio (SNR) for the weak diffuse component while avoiding detector saturation by intense Bragg peaks – a problem that has not been adequately addressed so far. Koch *et al.* (2021 ▸) considered various artifacts affecting single-crystal total scattering and developed data-processing protocols to mitigate them. Their focus, however, was on producing an artifact-free diffuse-scattering component, without addressing the accuracy of the Bragg-peak intensities. Moreover, some artifacts, such as blooming, which are relevant for the amorphous silicon area detector used in their study, become less significant for the single-photon-counting hybrid pixel detectors increasingly adopted at synchrotron facilities.

In essence, combining integrated Bragg peak intensities and 3D diffuse scattering from a single crystal in structural refinements does not require both components to be in the same dataset. From a practical standpoint, it would be computationally more effective to introduce them separately, with Bragg peaks included to large momentum transfers to provide sensitivity to atomic probability density distributions, and diffuse intensities over more limited reciprocal-space volumes sufficient to uncover correlations but amenable to fitting using current computing power.

Regardless of the analysis approach, quantitative fitting of diffuse scattering, whether in reciprocal space or after the Fourier transform, requires placing intensities on an absolute scale (*e.g.* electrons^2^ per atom). When Bragg peaks are acquired alongside the diffuse signal, they can be used to calibrate the scale by performing crystallographic refinements of the average structure. Alternatively, as has been shown recently (Eremenko *et al.*, 2025 ▸), one can exploit the asymptotic behavior of the scattering function obtained from the spherically integrated diffuse scattering to set the scale of diffuse scattering without Bragg intensities.

Here, we describe a workflow for collecting single-crystal X-ray total-scattering data using a synchrotron beamline optimized for the detection of weak diffuse scattering. We demonstrate an approach to address detector saturation due to Bragg peaks and to subtract Compton scattering, which contributes significantly to the scattering intensity at large momentum transfers. We further show the convergence of the two methods for scaling the intensity of diffuse scattering outlined above. Our validation of the approach, which relies on the known asymptote of the scattering function, enables robust scaling of single-crystal diffuse intensities, opening the door to a flexible combined analysis of Bragg and diffuse scattering even when these signals are collected on different instruments.

## 2. Experimental

### 2.1. Test system

In the absence of reference materials with well established atomic probability density distributions and interatomic correlations, we selected the canonical relaxor ferroelectric PbMg_1/3_Nb_2/3_O_3_ (PMN) (Bokov & Ye, 2006 ▸) as a test system because of the availability of extensive high-quality experimental data and results of large-box structural refinements from a combined fitting of these datasets (Eremenko *et al.*, 2019 ▸). PMN exhibits a relatively simple cubic perovskite average structure, but highly complex nanoscale correlations that generate prominent anisotropic diffuse scattering (*e.g.* Xu *et al.*, 2004 ▸). We used single crystals of PMN from our previous studies (Eremenko *et al.*, 2019 ▸; Eremenko *et al.*, 2025 ▸); the crystals did not display any noticeable radiation damage.

### 2.2. Synchrotron X-ray total-scattering measurements

X-ray scattering intensity from a PMN single crystal was measured at the diffraction side station of the ID28 beamline of the European Synchrotron Radiation Facility (Girard *et al.*, 2019 ▸). This station is equipped with a HUBER four-circle goniometer, a PILATUS^1^ 1M pixel area detector, and single-crystal X-ray diffractometry tools for crystal preparation and mounting. The measurements were conducted at ambient temperature using X-rays with a wavelength of 0.6968 Å. The approximately cylindrical crystal (∼30 µm in diameter) was glued to a glass needle and mounted on the sample holder. Scattering intensity was recorded in the ‘continuous’ mode, in which the crystal rotates at a constant angular speed around the diffractometer φ axis, integrating the detector images over a 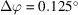 rotation step and the exposure time τ = 0.25 s. Full 360° rotations were repeated at four different detector angles, achieving a maximum scattering angle 2θ_max_ = 107° and momentum transfer *Q*_max_ ≈ 14 Å^−1^. The measurements were performed using six different primary-beam absorbers spanning five orders of magnitude in beam attenuation; for stronger absorbers, the full measurement was repeated two to four times to improve counting statistics. The degree of attenuation of the primary beam was determined for each absorber using a beam monitor. The experiment yields *J*_lab_(*x*_d_, *y*_d_, φ) representing the number of photons accumulated inside the detector pixel *x*_d_*y*_d_ during the crystal’s rotation over the angular range [φ, φ + Δφ] within the time interval τ and using the primary-beam absorber, corresponding to the beam monitor value *B*. The beam size of ∼50 × 50 µm was defined by the collimator. To ensure the same crystal volume within the beam throughout the experiment, we performed crystal-position scans after each change in the beam absorber.

Additional measurements without the sample indicated that the air-scattering contribution was of the order of a few percent of the diffuse intensity over the sampled *Q*-range. This contribution is small compared with other systematic uncertainties in the present scaling procedure. We therefore did not apply an explicit air-scattering correction and treat any residual contribution as part of the overall systematic uncertainty.

We used *CrysAlisPro* (Meyer, 2015 ▸) for the initial inspection of the scattering intensity and to determine the orientation matrix. The rest of the data analysis was performed using custom MATLAB scripts that provided the necessary flexibility and self-consistency. These scripts are supplied as supplementary information. The first stage of data processing involved determining the centers of mass of the Bragg reflections and refining the orientation matrices for datasets acquired with different beam absorbers. The differences between the obtained orientation matrices corresponded to the maximum strain 10^−3^ and rotation angle of 0.07°. During this refinement, a cubic lattice constraint was varied along with three Euler angles. Subsequently, we performed the reciprocal space reconstruction (RSR) as described below.

### 2.3. Laboratory X-ray diffraction measurements

A reference set of Bragg-peak intensities was collected with a Bruker D8 Venture diffractometer, where a small fragment of the PMN crystal, about 0.14 × 0.08 × 0.025 mm, was measured at ambient temperature (298 ± 2 K) using Mo *K*α radiation (0.71073 Å). The integrated intensities were obtained in the Bruker *APEX5* software and corrected for absorption using *SADABS* (Krause *et al.*, 2015 ▸). The minimum and maximum transmission values were 0.121 and 0.266, respectively. The measurements covered the *hkl* range of −8 ≤ *h*, *k*, *l* ≤ 8 out to *Q*_max_ = 12.9 Å^−1^, yielding a total of 7463 reflections, of which 90 unique reflections (*R*_int_ = 0.032, *R*_sig_ = 0.0154) were used in the structural analysis.

## 3. Methodology for processing 3D total-scattering data

**Figure 1:**
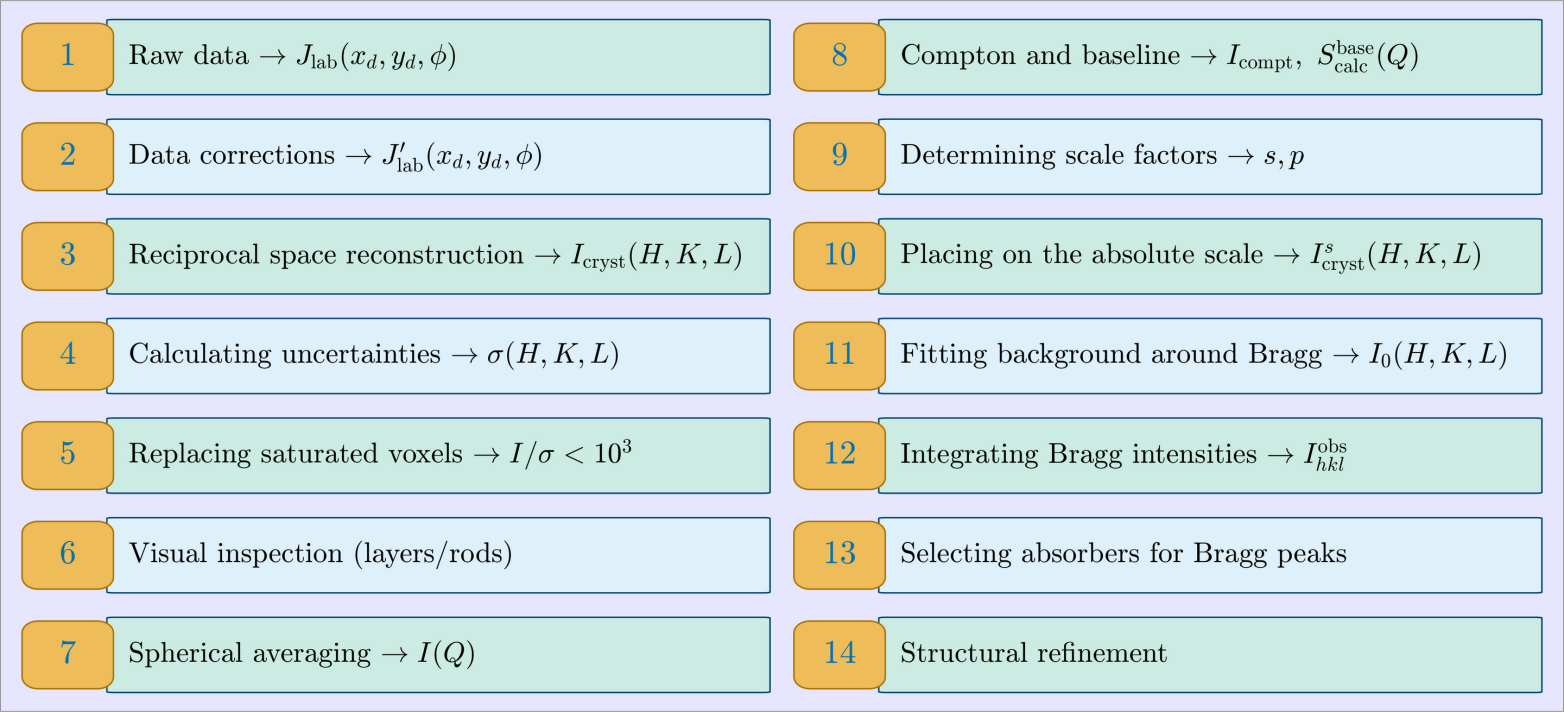
Workflow for converting raw X-ray scattering intensities into absolute-scale data. The intensity notations correspond to those used in the equations presented in the subsequent sections.

Fig. 1 ▸ presents a workflow that transforms raw intensities into absolute-scale scattering data. The following sections detail each step.

### 3.1. Reciprocal space reconstruction

**Figure 2:**
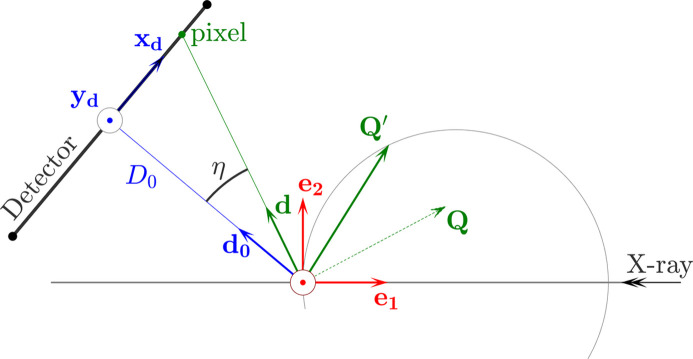
Schematic illustration of the diffraction experiment, introducing notations used in the text. The image shows the primary X-ray beam, Cartesian laboratory coordinate system **e**_1_**e**_2_ (**e**_3_ is defined as their cross product). **d**_0_ and **d** are the unit vectors defining the direction of the detector normal and the direction from the crystal to the given detector pixel, respectively. The angle between these two vectors is defined as the oblique incidence angle η. *D*_0_ is the distance to the detector plane. **x**_d_, **y**_d_ are the unit vectors defining the detector axes. **Q** and **Q**′ are the scattering vectors before and after rotation.

The first objective of RSR is to transform the instrument-related coordinates *x*_d_, *y*_d_, φ into the components of the scattering vector **Q** (Fig. 2 ▸) and to convert the measured scattering intensity *J*_lab_(*x*_d_, *y*_d_, φ) into the *I*(*Q*_*x*_, *Q*_*y*_, *Q*_*z*_) = *I*_cryst_(*H*, *K*, *L*) look-up tables. Here, *Q*_*x*_, *Q*_*y*_, *Q*_*z*_ / *H*, *K*, *L* denote the components of the scattering vector in either the Cartesian laboratory **e**_*i*_ or the reciprocal crystallographic 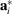 coordinate systems (see Appendix *A*[App appa] and Fig. 2 ▸ for more details) and at zero diffractometer angles:
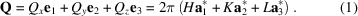


The second objective is to correct *J*_lab_(*x*_d_, *y*_d_, φ), enabling its conversion to a form that can be modeled using coherent scattering intensity *I*_coh_(**Q**), which can be expressed as
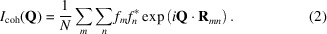
Here, *N* is the total number of atoms in the irradiated volume of the crystal, *f*_*m*_ = *f*_*m*_(*Q*) are the atomic scattering factors, and **R**_*mn*_ = **R**_*m*_ − **R**_*n*_ is the vector connecting atoms *n* and *m*.

Our RSR implements the necessary data correction and normalization steps according to the kinematical theory of X-ray scattering (Warren, 1990 ▸; Guinier, 1994 ▸). The first step applies pixel-by-pixel (polarization, Lorentz and oblique incidence) and flat-field (exposure time τ, primary beam monitor *B* and the distance to the detector *D*_0_) corrections:
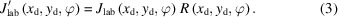
The correction factor *R* is introduced as (see Appendix *B*[App appb] for more details)
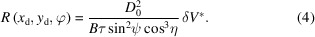
Here, ψ = ψ(*x*_d_, *y*_d_) is the angle between the polarization direction of the primary beam and the propagation direction of the scattered beam and η = η(*x*_d_, *y*_d_) is the oblique incidence angle (Fig. 2 ▸). δV* = δV*(*x*_d_, *y*_d_, φ) is the reciprocal space volume covered by the single 3D pixel, which can be calculated from the known functional dependence *Q*_*x*, *y*, *z*_(*x*, *y*, φ) (see Appendix *A*[App appa] for more details):
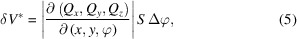
where *S* is the area of the detector pixel.

The next RSR step partitions reciprocal space into fixed-size voxels extending over the Δ*H*, Δ*K* and Δ*L* reciprocal lattice units (r.l.u.) along 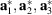, respectively. We assume that the volume of the voxel is significantly greater than δV*. The reconstruction algorithm converts *Q*_*x*_, *Q*_*y*_, *Q*_*z*_ into *H*, *K*, *L* and assigns all measured pixels to their corresponding voxels. At this stage, we also symmetrized the data using the operations of the 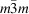 point group, generating symmetric copies of each pixel in the *H*, *K*, *L* space. This symmetrization naturally compensates for missing intensity values arising from the intermodular gaps of the PILATUS detector and improves the robustness of the diffuse-scattering subtraction procedure.

We then accumulate the volume-average voxel intensities *I*_cryst_(*H*, *K*, *L*) according to
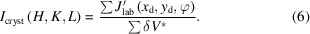
Here, both summations are over all pixels that fall into the *HKL*-based voxel. Appendix *B*[App appb] shows that these reconstructed values are related to the scattering amplitude as
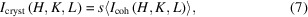
where *s* is the scaling constant and the average is over the *HKL*-based voxel. Approaches to determining this constant will be considered in the next sections.

Our RSR procedure enables calculating the standard uncertainties σ(*H*, *K*, *L*) of the *I*_cryst_(*H*, *K*, *L*) values, assuming Poisson counting statistics so that σ^2^(*x*_d_, *y*_d_, φ) = *J*_lab_(*x*_d_, *y*_d_, φ). Then, according to equations (3) and (6)
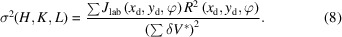


### 3.2. The problem of detector oversaturation

The simultaneous acquisition of both Bragg and diffuse-scattering intensities may lead to saturation artifacts in certain pixels *J*_lab_(*x*_d_, *y*_d_, φ), particularly in the proximity of intense Bragg reflections. Moreover, any departure from the linear response of the detector leads to a systematic underestimation of recorded intensity values. The PILATUS active pixel area detector is known to maintain linearity at a photon count rate below 10^6^ cps. This quantity is defined by the deadtime of the detector. Additionally, a hard cap of  1.5 × 10^6^ counts per pixel per frame is imposed by the dynamic range of the detector. A pixel is therefore considered oversaturated if either of these limits is exceeded.

In our experimental setup, each frame was acquired with a minimum exposure time of 0.25 s. Thus, we conservatively define a threshold of 10^5^ counts per pixel per frame, corresponding to an SNR ∼ 300, as a safe upper limit to ensure unsaturated signal acquisition, assuming that the intensity does not vary significantly within a pixel.

If the latter is true, an intuitive approach to correcting oversaturation is to replace saturated pixels with equivalent ones from the dataset measured using an appropriate absorber. However, this approach is not feasible in practice. First, small discrepancies in the refined orientation matrices during successive measurements lead to different reciprocal space coordinates *HKL* assigned to the same detector pixel. More importantly, in the vicinity of Bragg peaks, the scattered intensity varies rapidly within a pixel over the rotation interval [φ, φ + Δφ]. As a result, even if the recorded (integrated) pixel intensity remains below the threshold, the instantaneous count rate may exceed the count rate threshold, making it impossible to reliably identify saturated pixels on an individual basis.

For these reasons, we perform the correction at the voxel level rather than for individual pixels. Each voxel represents a volumetric element in reciprocal space that aggregates the intensity contribution of 10^3^ to 10^4^ pixels. For voxel-based reconstructions, we define a higher saturation threshold corresponding to SNR ∼ 1000, providing a robust criterion for identifying and mitigating saturation.

**Figure 3:**
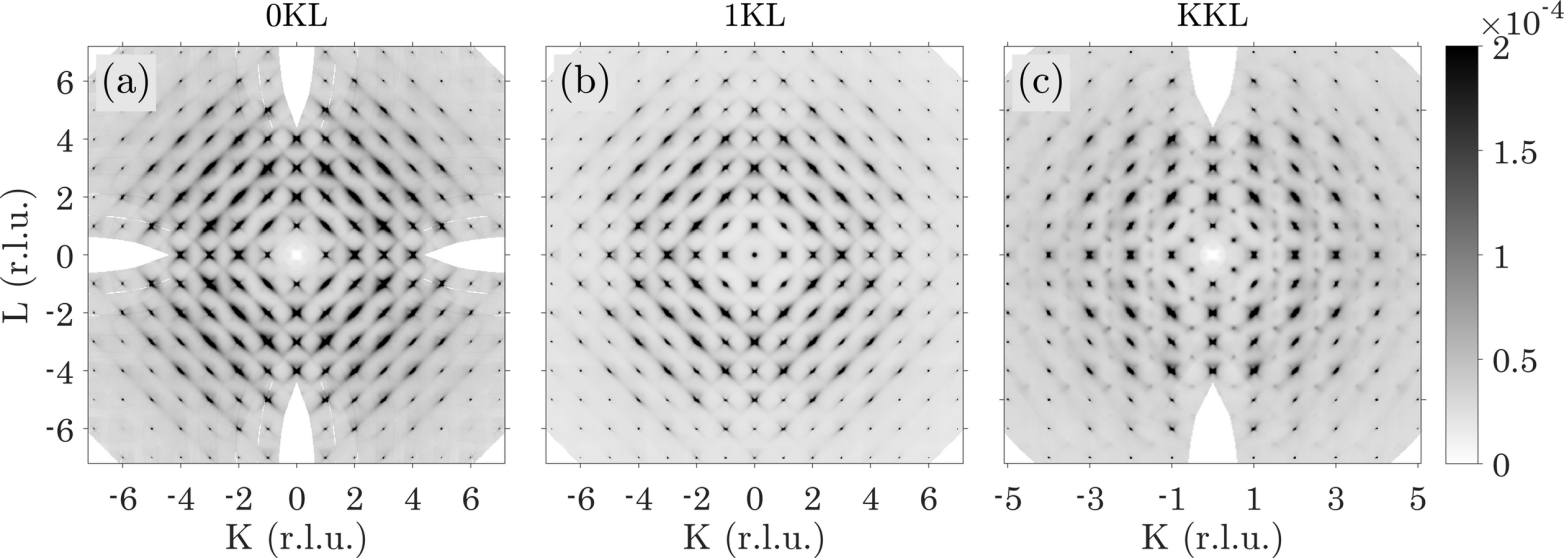
Grayscale maps of 2D reconstructions of X-ray scattering intensity in the (*a*) 0*KL*, (*b*) 1*KL* and (*c*) *KKL* layers obtained from the combined (SNR < 1000) dataset, each integrated over the range of 0.05 r.l.u. The maps reveal both sharp Bragg peaks and strongly anisotropic diffuse scattering. Due to the extremely high and typically oversaturated intensity of the Bragg peaks, the grayscale was clipped at 0.0002 of the maximum intensity to enhance the visibility of the diffuse features.

**Figure 4:**
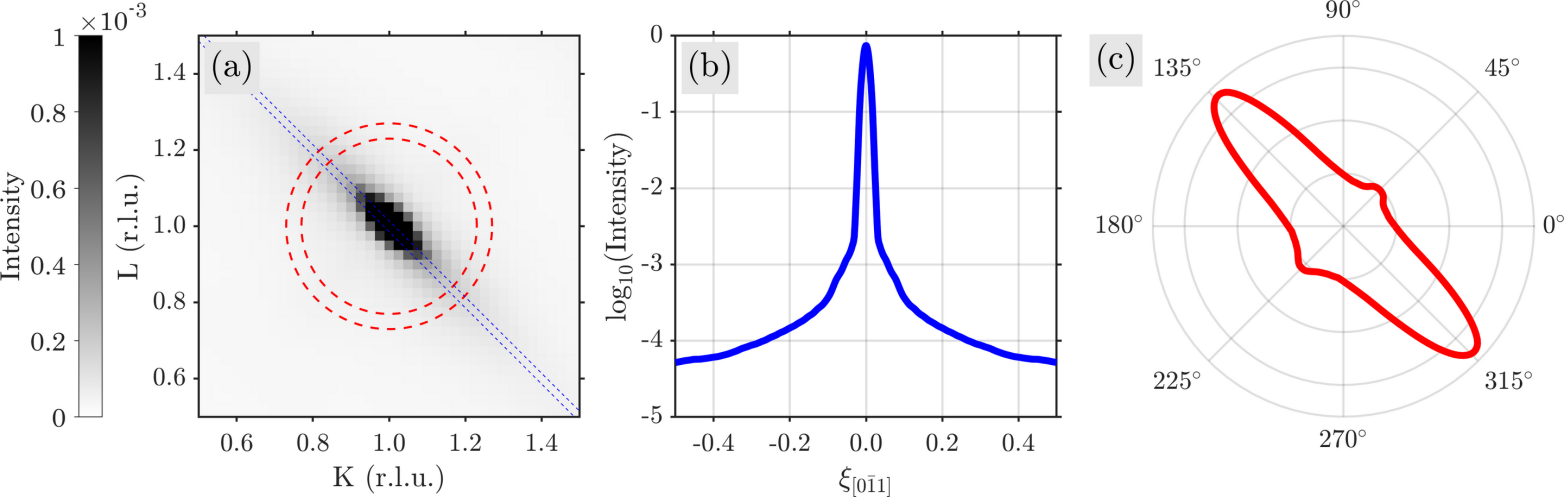
Details of the diffuse-scattering intensity around the 011 Bragg reflection from the combined dataset (SNR < 1000 criterion). The figure consists of three panels that present complementary aspects of the reconstructed intensity distribution in the 0*KL* reciprocal lattice plane. (*a*) Reconstructed 2D intensity map around the Bragg peak, displayed over ±0.5 r.l.u. The red circular dashed region marks the radial zone used for angular averaging, and blue dashed lines indicate the strip used to extract a one-dimensional profile along the diagonal direction. (*b*) Logarithmic intensity profile along the [0*K**K*]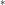 reciprocal direction, averaged over a finite-width strip perpendicular to the path. (*c*) Polar plot of the angular dependence of the diffuse intensity, extracted from the region shown in (*a*).

This approach is well suited for diffuse scattering, where the intensity varies slowly over both pixel and voxel dimensions. By combining datasets collected with different absorbers, we construct a lookup table in which the diffuse component is effectively free from saturation artifacts. Figs. 3 ▸ and 4 ▸ present the results. Fig. 3 ▸ displays the two-dimensional reconstruction of 0*KL*, 1*KL* and *KKL* layers, each integrated over the range of 0.05 r.l.u. Fig. 4 ▸ provides additional details on the scattering intensity, including its radial and angular dependence as shown in the intensity profile along the cubic face diagonal direction and the polar plot illustrating the anisotropy of the distribution.

This voxel-level criterion becomes unreliable in the vicinity of Bragg peaks, where the photon flux is highly localized within angular regions much smaller than a voxel. In such cases, saturation cannot be reliably detected from voxel-averaged intensities. Therefore, the procedure described above is applied only to diffuse scattering, while Bragg-peak intensities are treated separately, as described in Section 3.4.

### 3.3. Placing data on the absolute scale

In traditional crystallographic refinements, the scale factor *s*_B_ is treated as an independent variable, obtained by minimizing the difference between 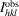 and 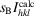. Here, 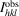 is the integrated intensity of the *hkl* Bragg reflection, and 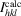 is defined according to
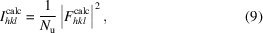
where
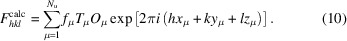
*N*_u_ is the number of atoms in the crystallographic unit cell. 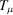 are the Debye–Waller factors associated with atomic sites having coordinates 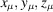. 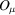 are the occupancies of these sites. It is possible to show [see *e.g.* Warren (1990 ▸)] that
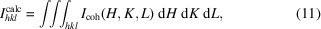
where the integration is carried out over the Bragg peak *hkl*.

In contrast, refinements based on total-scattering data using large atomic configurations require the experimental data to be on an absolute scale (electrons^2^ per atom) so that no scale factor is involved in the fit. Although the scale factor can, in principle, be treated as a variable, in large-configuration refinements with many degrees of freedom (*e.g.* RMC-type approaches), it becomes strongly coupled with the amplitudes of occupational and displacement correlations. Diffuse intensities generally scale with the square of these correlation amplitudes. Therefore, fitting procedures such as those involving the RMC method will ‘use’ a scale factor to compensate for correlation strength, potentially leading to significantly biased structural parameters (Eremenko *et al.*, 2019 ▸; Eremenko *et al.*, 2025 ▸; Krayzman *et al.*, 2022 ▸). While physical constraints encoded in the data, such as the decay of correlations with distance, reduce this ambiguity, they are often insufficient to drive the fit to the correct solution. For this reason, placing diffuse-scattering data on an absolute scale prior to refinements provides an important external constraint and improves the reliability of the derived structural information.

If both Bragg and diffuse scattering are measured simultaneously, this issue can be resolved by first performing a conventional crystallographic refinement to determine *s*_B_ and subsequently rescaling the entire total-scattering dataset, including its diffuse component. Alternatively, the scattering data can be placed on an absolute scale by exploiting the known asymptotic behavior of the powder scattering function, as suggested by Eremenko *et al.* (2025 ▸). In this approach, the 3D total-scattering data are first spherically averaged and converted into the scattering function, which is then matched to its theoretical baseline calculated from the composition and atomic displacement parameters (*U*_*ij*_). These parameters need only be known approximately and can be adopted from the literature, if available, or estimated from crystallographic refinements using Bragg intensities, either from the same total-scattering dataset or from a separate experiment. Here, we demonstrate that the two scaling methods converge, yielding similar outcomes.

**Figure 5:**
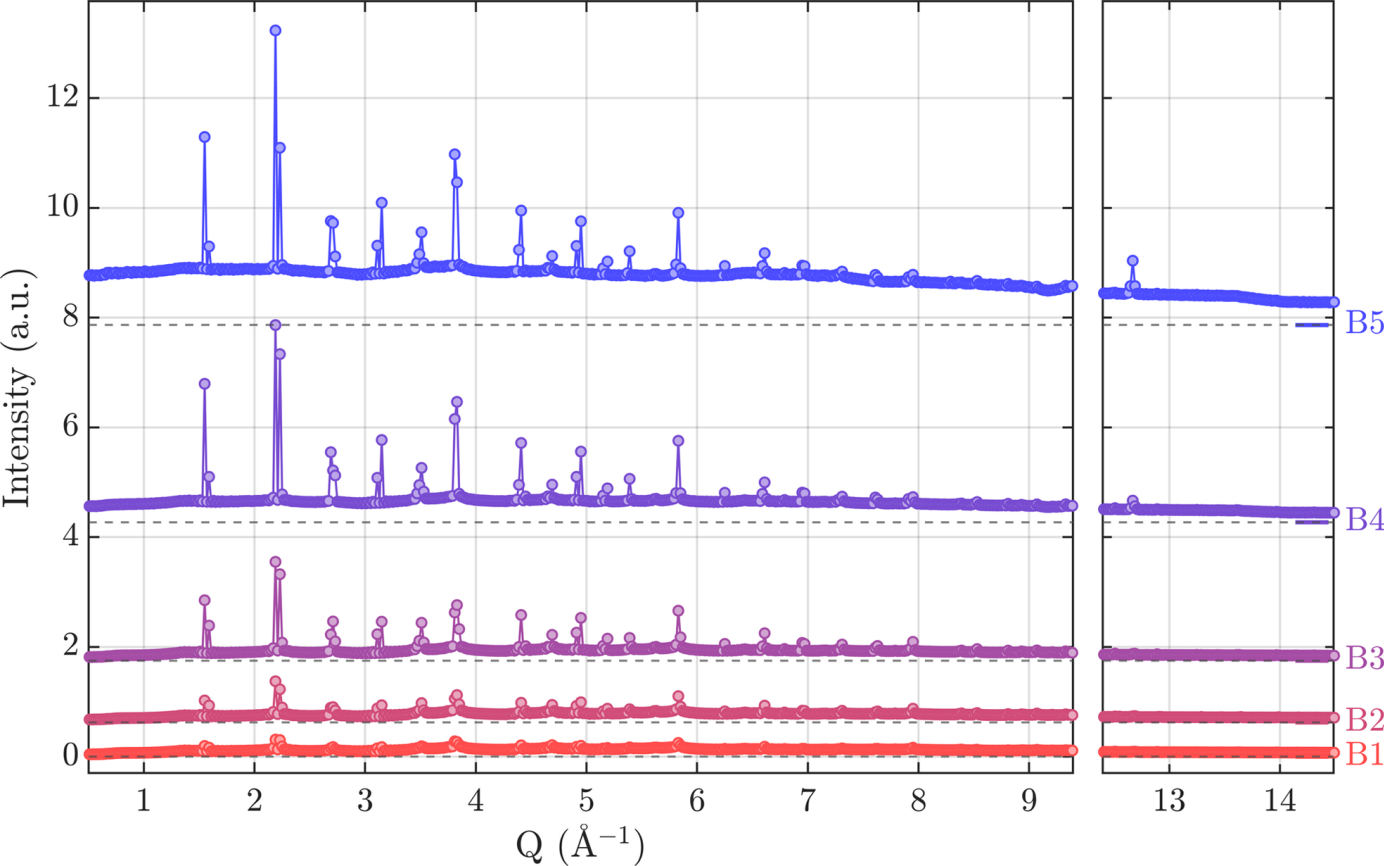
*I*(*Q*) according to equation (12) for five absorbers (B1–B5, from bottom to top), vertically offset for clarity. In this series, B1 corresponds to the weakest absorber and B5 to the strongest. Horizontal dashed lines indicate the zero-intensity baseline for each curve. The absorber used for each dataset is indicated on the right. According to equations (3), (4) and (12), all intensities are normalized to the beam monitor. The figure illustrates saturation effects in Bragg peaks, which result in underestimated intensities when weaker absorbers are used. It also highlights artifacts in the diffuse background arising from limited counting statistics in measurements with stronger absorbers.

We first present the scaling approach based on the asymptotic behavior of the powder scattering function. The procedure includes the following steps:

*Step 1.* Integrating the 3D scattering intensity distribution *I*_cryst_(*H*, *K*, *L*) ≡ *I*(**Q**) over all possible crystal orientations to obtain a 1D intensity function *I*(*Q*) that would be measured for an equivalent powder specimen:
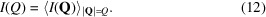
In practice, this integration is performed by dividing the *Q*-range of interest into a finite number of bins and averaging all *I*(**Q**) values corresponding to each |**Q**| within a given bin. Fig. 5 ▸ displays the result of such averaging for datasets collected with five different beam absorbers. The graphs are vertically offset for clarity, and the zero level for each graph is indicated by a dashed line and a corresponding label specified on the right-hand side.

*Step 2*. Introducing the calculated and observed scattering functions.

The calculated scattering function can be expressed (*e.g.* Farrow & Billinge, 2009 ▸) as
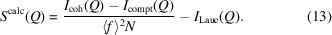
Here, *I*_coh_(*Q*) is the spherically averaged coherent scattering intensity, *I*_compt_(*Q*) represents the inelastic Compton scattering and *I*_Laue_(*Q*) is the monotonic (Laue) diffuse scattering, which in a multicomponent system is expressed as
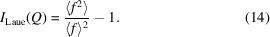
We define the *observed* scattering function *S*^obs^(*Q*) as
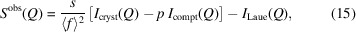
where *s* are *p* are the scaling coefficients. *I*_compt_(*Q*) is calculated according to Bikondoa & Carbone (2021 ▸) as described in Appendix *C*[App appc].

*Step 3:* Determination of the scale coefficients *s* and *p.*

The function *S*^calc^(*Q*) asymptotically approaches unity at high *Q*. Thus, *s* and *p* can be determined by imposing the same requirement on *S*^obs^(*Q*). Measurements have to extend to *Q* values sufficiently large for *S*(*Q*) to attain this asymptote – a condition that can be verified by considering the expected baseline of the scattering function, 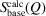:
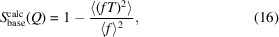
where
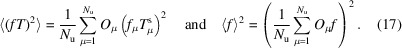
Here, 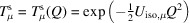 is the orientational average of the generally anisotropic Debye–Waller factor, 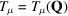, and *U*_iso,μ_ is the equivalent isotropic atomic displacement parameter for site μ. Calculating 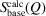 requires *U*_iso,μ_ and 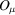 only. The supporting information for this article includes a script that calculates 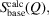 given the chemical composition and *U*_*ij*_ tensors.

In our case, the conditions were implemented by selecting all data points with *Q* > *Q*_min_ = 12 Å^−1^ and using linear regression for matching *S*^obs^(*Q*) to 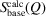 to find *s* and *p*. Then, we normalized our reconstructed total intensities per atom according to
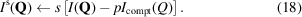


**Figure 6:**
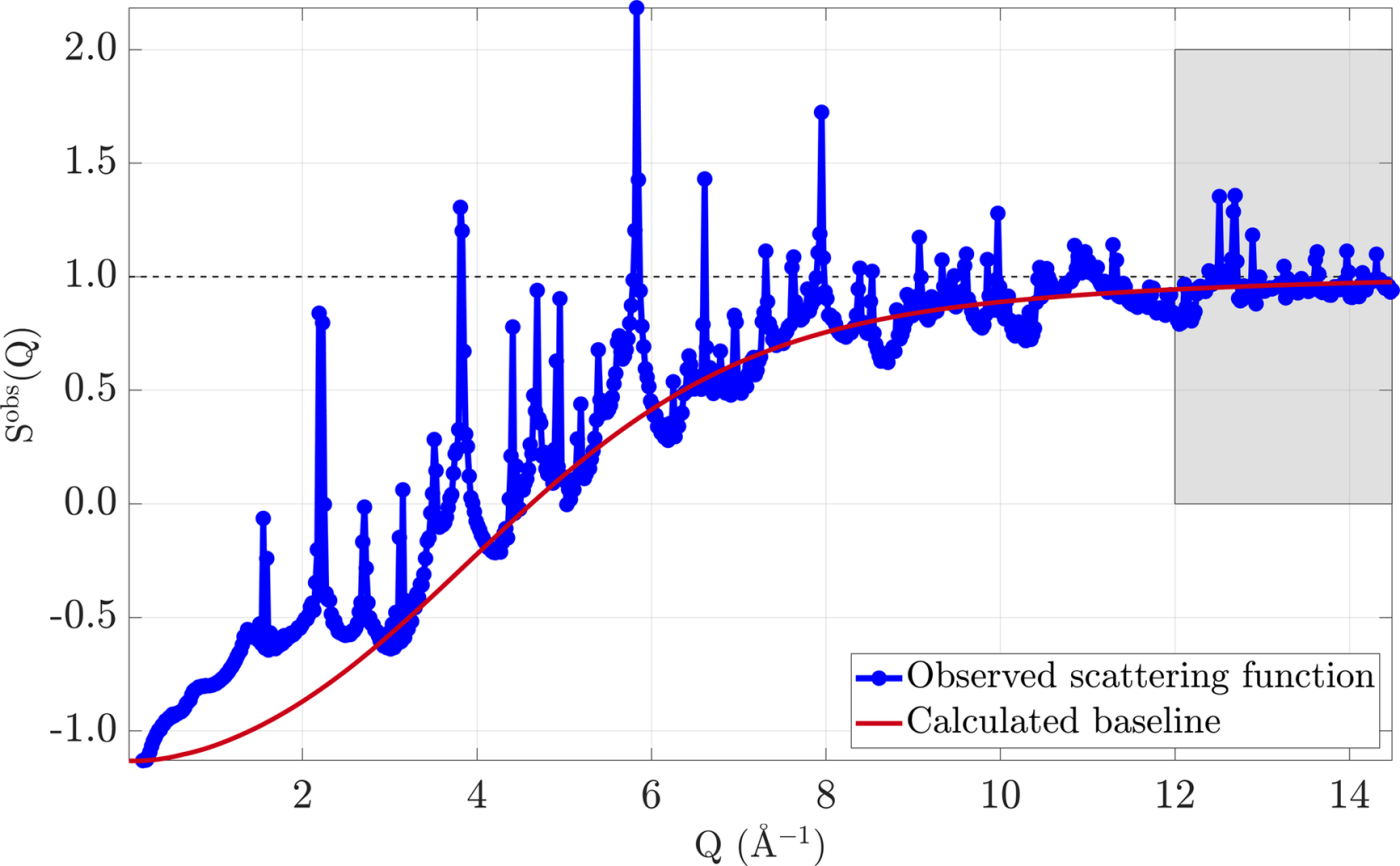
Normalized scattering function *S*^obs^(*Q*) as defined in equation (15), where coefficients *s* and *p* were calculated so that the resulting *S*^obs^(*Q*) asymptotically approaches 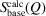 at high *Q.* The rectangle on the right highlights the *Q*-range that was used to determine these coefficients. The red line shows the baseline, calculated according to equations (16) and (17).

Fig. 6 ▸ shows the resulting scattering function, determined according to equations (16) and (18). The same figure also shows the baseline 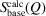. The interval *Q* > *Q*_min_ which was used for linear regression to obtain *s* and *p* is highlighted with a rectangle.

The relative contribution of Compton scattering to the total intensity can reach ∼50% at *Q* = 14 Å^−1^ (as shown in Fig. 9 in Appendix *C*[App appc]). Subtracting this parasitic background is essential for the quantitative fitting of diffuse intensities to *Q* > 5 Å^−1^.

To assess the effect of the choice of *Q*_min_ on *s* and *p*, we repeated the procedure for a range of *Q*_min_ values between 7 and 12 Å^−1^, while keeping *Q*_max_ fixed. The resulting dependencies *s*(*Q*_min_) and *p*(*Q*_min_) are shown in Appendix *D*[App appd]. The corresponding variations reflect both the statistical uncertainty in the *I*(*Q*) values and the systematic uncertainty associated with the scaling procedure. From the maximum relative deviation of *s* and *p* over this *Q*_min_ range, we estimate their uncertainties not to exceed 6%, yielding *s* = 0.034 (2) and *p* = 43 (3).

### 3.4. Determining Bragg intensities

The RSR process does not distinguish between Bragg and diffuse scattering. However, combining these signals in a single lookup table poses challenges that must be addressed if fitting this table as a single data set. The difficulty arises because Bragg peaks are typically confined to a single detector pixel and have angular widths that are one to two orders of magnitude smaller than Δφ. As a result, experimental data provide Bragg-peak intensities integrated over the corresponding voxel volumes.

Below, we describe a procedure for extracting observed Bragg intensities 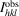 that can be matched to 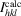 calculated from a structural model according to equations (9) and (10). The first step of the proposed procedure involves subtracting the diffuse-scattering background *I*_0_(*H*, *K*, *L*) from the total reconstructed intensity *I*_cryst_(*H*, *K*, *L*). At this stage, the background intensity *I*_0_(*H*, *K*, *L*) beneath the Bragg reflection is estimated by fitting a 3D pseudo-Voigt function to the portion of *I*_cryst _(*H*, *K*, *L*) located at a distance greater than 0.06 r.l.u. from the peak center. Our use of a 3D pseudo-Voigt function does not represent a physically rigorous model of diffuse scattering. We employ it as a flexible, empirical function to approximate the smooth background intensity, enabling separation of Bragg and diffuse contributions as required to address the goals of this study. The extracted Bragg-peak intensity values were found to be insensitive to small variations in the pseudo-Voigt parameters within the fitting uncertainty. The uncertainty associated with the diffuse-background approximation is therefore implicitly included in the uncertainty assigned to the extracted Bragg-peak intensities and propagates into the final scale-factor determination.

**Figure 7:**
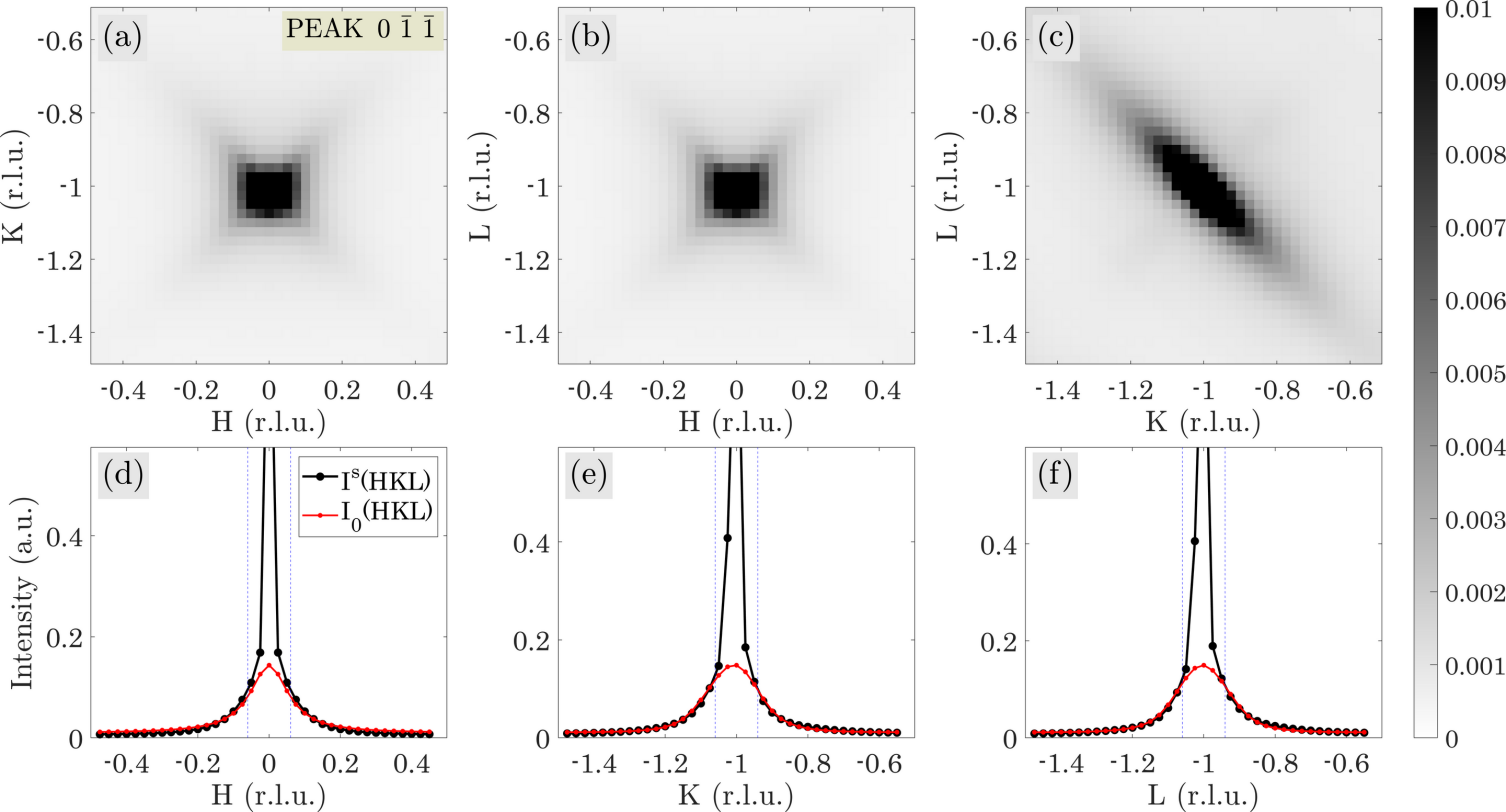
The reconstructed intensity 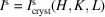 around the 011 Bragg reflection. The top panels show *HK*, *HL* and *KL* projections, while the bottom panels show the *H*, *K* and *L* dependencies, along with the fitted background curve *I*_0_(*H*, *K*, *L*).

Fig. 7 ▸ illustrates this background subtraction procedure. It represents a 3D reconstruction of *I*_cryst _(*H*, *K*, *L*) around the 011 reflection, including the *HK*, *HL*, *KL* projections (integrated along the missing coordinate in the range of 0.05 r.l.u.) and *H*, *K* and *L* intensity profiles (integrated over the two missing coordinates). Each 1D plot also includes its corresponding fitted background profile *I*_0_(*H*, *K*, *L*). Then the observed Bragg intensities are calculated as follows:
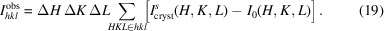
Here, the product Δ*H* Δ*K* Δ*L* defines the voxel volume. This factor is introduced to account for the reciprocal space integration as defined in equation (11). The summation is performed over those voxels in the reciprocal unit cell centered on the reciprocal lattice node *hkl* that fall within the range (|*H* − *h*|, |*K* − *k*|, |*L* − *l*|) < 0.5.

We use equation (19) to generate tables of 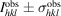 for each measured reflection and each absorber. We then examine these intensities versus the beam monitor value *B*. Because the RSR normalizes the intensities by *B* [equations (3) and (4)], unsaturated reflections are flat with respect to *B*. In contrast, saturated reflections display underestimated intensities at large *B*, where the primary beam is more intense.

**Figure 8:**
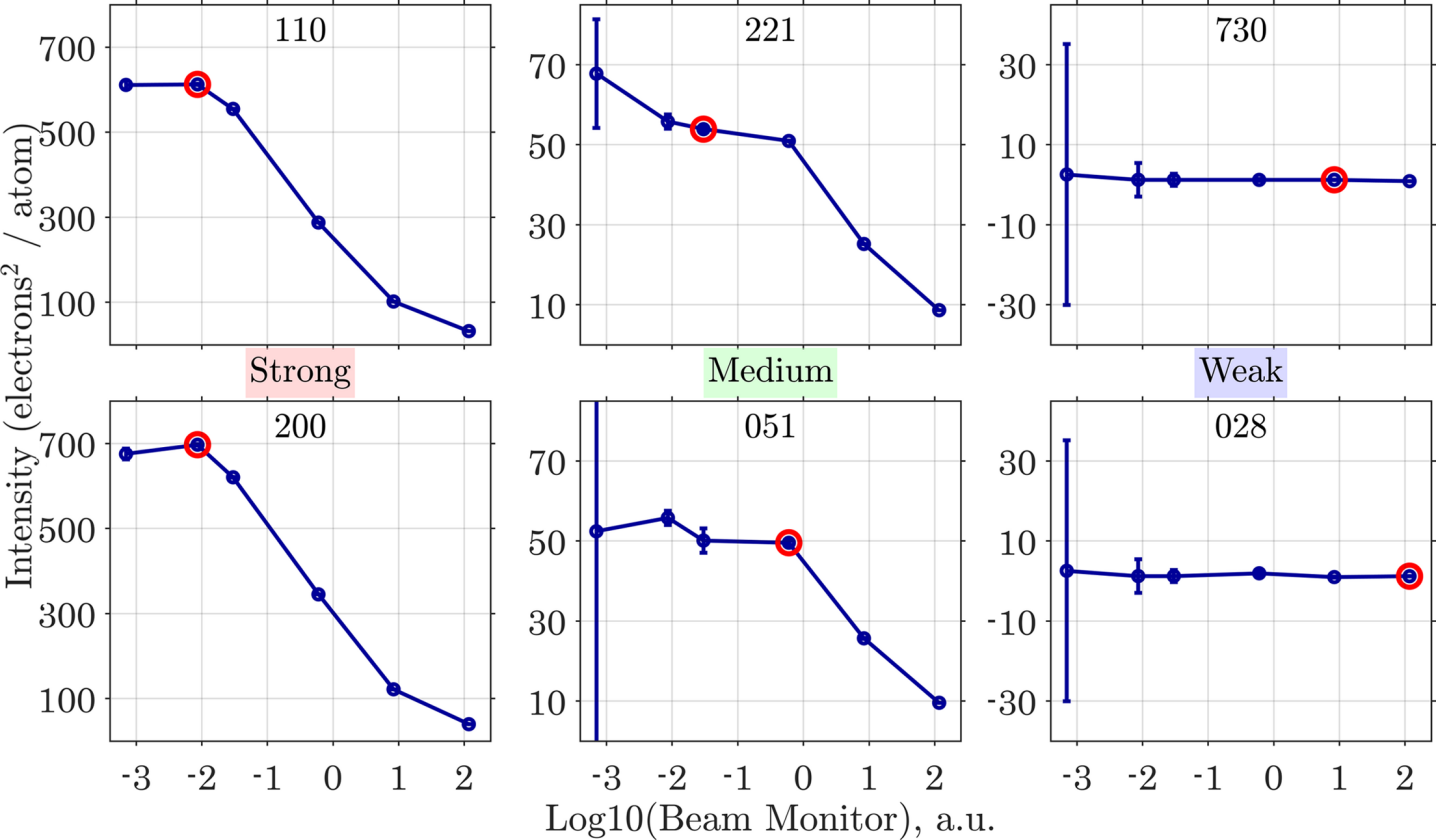
Dependence of reflection intensities on the primary-beam monitor value *B*, shown on a logarithmic scale, for three groups of representative reflections: strong (110, 200), medium (221, 051) and weak (730, 028). Each panel displays the integrated intensity 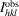 with error bars as a function of 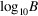. For strong reflections, intensities decrease at higher beam-monitor values due to detector saturation. Conversely, weak reflections exhibit low signal-to-noise ratios at low flux. Red circles indicate the absorber setting automatically selected for each reflection by the procedure used to optimize data quality for structural refinement.

**Figure 9:**
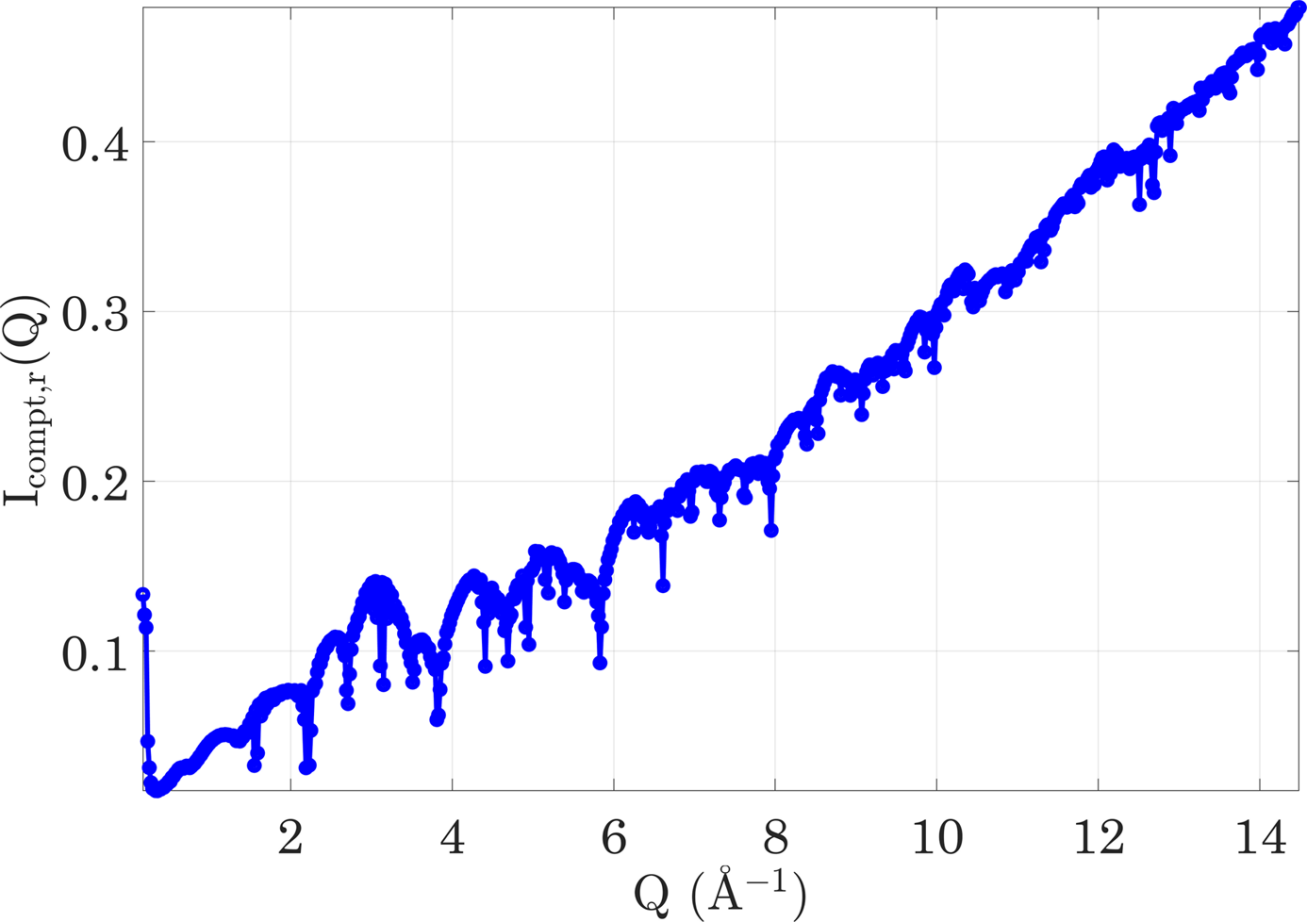
*Q* dependence of Compton scattering contribution defined as *I*_compt,r_(*Q*) =*pI*_compt_(*Q*)/*I*(*Q*). The graph shows that at high *Q* values Compton scattering can contribute significantly — up to 50% to the total scattering.

Fig. 8 ▸ plots the examples of 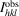 versus *B* for three reflection groups: strong (110 and 200), medium (221 and 051) and weak (730 and 028). As expected, strong peaks exhibit reduced intensities at large *B* due to detector saturation; for these reflections, we select intensities from the datasets recorded with a higher-attenuation absorber. Conversely, weak reflections require higher-flux data to achieve adequate SNR. We automated this reflection choice by inspecting 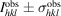 as a function of *B* using the procedure below. For each reflection, this procedure selects the highest-intensity measurement that is not saturated while maintaining sufficient counting statistics.

(*a*) Starting from the weakest absorber *B*_1_, we compare successive intensity values 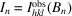 and their uncertainties 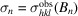.

(*b*) If the condition *I*_*n*+1_ − *I*_*n*_ > (σ_*n*+1_ + σ_*n*_) is satisfied, the value *I*_*n*_ is considered affected by saturation and is replaced with *I*_*n*+1_.

(*c*) The process is repeated iteratively until no further replacements are required or statistically significant values 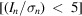 are exhausted.

In Fig. 8 ▸, red circles mark the absorber selected for each reflection. The intensity values for those voxels in the total-scattering dataset that contain Bragg peaks can now be replaced with their corresponding unsaturated values.

## 4. Crystallographic refinements

All refinements were performed in *ShelXle* (Hübschle *et al.*, 2011 ▸; Sheldrick, 2015 ▸). We considered two Bragg intensity datasets 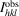: one from the laboratory instrument, with a proven track record of providing reliable structural models, and another from our synchrotron measurements, with intensities extracted as described in Section 3.4. The initial model assumed a perovskite *Pm*3*m* space group with all the atoms residing at the ideal positions: Pb @ 1*a* 000; Mg and Nb @ 1*b* ½½½; O @ 3*c* ½½0. This model produced a poor fit and anomalously large atomic displacement parameter for Pb for both datasets (laboratory data: *wR*2 ≃ 16%, *U*_Pb_ ≃ 0.08 Å^2^; synchrotron data: *wR*2 ≃ 20%, *U*_Pb_ ≃ 0.08 Å^2^).

We then considered two models with Pb disordered over multiple sites offset from the ideal central position. One assumed Pb atoms shifted along the 〈100〉 directions (Wyckoff position 6*e:x* 0 0) and the other assumed Pb atoms shifted along the 〈111〉 directions (Wyckoff position 8*g*: *x* *x* *x*). Both split-site models produced a significant improvement in fit with similar agreement factors for each dataset. For the laboratory data, *wR*2 ≈ 7.2% and *wR*2 ≈ 6.98% for the eight- and six-site Pb models, respectively. Regardless of the displacement directions, the magnitude of the Pb off-centering relative to the centrosymmetric positions was ∼0.28 Å. For the synchrotron data, the agreement factors were considerably worse, with *wR*2 ≈ 9.2% for both eight-site and six-site models. Refining the Nb fraction ratio using the laboratory data yielded 0.652 (14), which is close to the expected value of 0.667. For the synchrotron data, the refined fraction of Nb deviated significantly from the expected value (*i.e.* 0.5 instead of 0.667); therefore, in the final refinement, we kept the composition fixed at the stoichiometric value. The six- and eight-site models were indistinguishable, in line with the results reported by Eremenko *et al.* (2025 ▸), who observed the coexistence of both types of Pb displacements.

Despite the significantly poorer agreement factors for the synchrotron data, the structural parameters, including the magnitude of the Pb off-centering and the *U*_*ij*_ values for Mg/Nb and O sites, were similar to those obtained from the laboratory data (Table 1 ▸), indicating that the structural parameters, in our case, are robust, even in the presence of apparently larger systematic errors. The presence of larger errors in the synchrotron data was also reflected in the relatively large extinction coefficient (1.34), compared with the much more reasonable value of 0.35 obtained from fitting the laboratory dataset.

## 5. Scale factor

Given that the synchrotron datasets 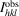 were placed on the absolute scale (per atom), the expected scale factor *s*_B_ that matches 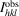 and 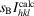 is 1. Accordingly, the scale factor *s*_F_ that matches 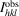 and 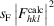 (this is how the scale factor is defined in *ShelXle*) is expected to be equal to 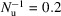 [equation (9)]. Our refinement yields *s*_F_ ≈ 0.35. Despite the ∼1.75-fold difference, we regard the agreement as reasonable given the experimental limitations, the strong scale–extinction correlation and the complexity of data processing. This result validates the scaling procedure based on the asymptotic behavior of the scattering function.

Indeed, while the discrepancy of 1.75× may appear significant, in our experience, allowing the scale factor to vary freely in RMC refinements involving diffuse scattering often yields values that deviate by an order of magnitude or more from the absolute scale. The reason is the intrinsic coupling between scale and correlation amplitudes during fitting, as discussed in Section 3.3. By contrast, a discrepancy of ∼1.75× is likely comparable to the uncertainties arising from the various approximations inherent in the extraction of structural information from diffuse scattering.

In *ShelXle* refinements, the scale factor is strongly correlated with the extinction coefficient. In our case, for the synchrotron data, the correlation coefficient between these two parameters was ∼0.82. For the laboratory data, it was even larger (∼0.92). Despite this strong correlation, the refinements still show a well defined minimum in the agreement factor (*R*1), as illustrated in Fig. S1 (supporting information), at the scale value close to the reported ∼0.35. The actual uncertainties in the resulting structural parameters, reflecting the combined effect of multiple error sources, including the scale–extinction correlation, can be assessed from Table 1 ▸, which compares refinements of the same model using data collected on different instruments. In the present case, the structural parameters appear to be reasonably robust.

Overall, a separate scaling procedure based on the predicted baseline of the scattering function provides a check on the scale factor, improving the reliability of scaled intensities, both Bragg and diffuse. While using Bragg and diffuse components obtained from the same measurements carries obvious advantages, especially when performing *in situ* measurements, the availability of a validated scaling procedure just for the diffuse part permits combining Bragg and diffuse datasets measured using different instruments.

## 6. Summary

We developed a procedure to obtain Bragg and diffuse X-ray scattering intensities from a single 3D dataset. Experimental measurements were performed using a synchrotron beamline optimized for recording weak diffuse scattering. The currently unavoidable detector saturation caused by strong Bragg reflections was addressed by collecting data with a series of absorbers spanning five decades in intensity. We developed robust protocols to identify voxels in the reconstructed 3D intensity distributions that were affected by saturation and replace their intensities with those from datasets collected using the appropriate absorber.

We then compared two scaling methods for relating measured intensities to their absolute-scale (electrons^2^ per atom) theoretical values. The first method uses traditional crystallographic refinements, matching Bragg intensities calculated for the average structure model to the observed values. The second, introduced recently but still unverified, matches the 1D scattering function, calculated as a spherical average of the measured scattered intensities, to a theoretical baseline estimated from the chemical composition and atomic displacement parameters. The latter function has a well established behavior at large momentum transfers, which can be used to scale experimental data; this scaling can be performed on diffuse scattering alone, without including Bragg reflections. The two methods yielded reasonably consistent absolute scales, supporting the use of the calculated scattering-function baseline to scale the diffuse portion of the total signal.

With the ability to scale Bragg peaks and diffuse scattering independently, it is possible to combine these datasets in structural refinements even when they were measured with different instruments. For example, a viable approach would be to collect Bragg data using a laboratory diffractometer and diffuse-scattering data at a synchrotron or in an electron microscope. A capability for such refinements, using a list of observed structure factors and 3D diffuse scattering as input, has been implemented in *RMCProfile*, and examples will be reported separately.

## Supplementary Material

Crystal structure: contains datablock(s) global, I, II, III, IV. DOI: 10.1107/S1600576726006187/pag5004sup1.cif

Structure factors: contains datablock(s) I. DOI: 10.1107/S1600576726006187/pag5004IIsup2.hkl

Supplementary software packages for reciprocal-space intensity reconstruction (MATLAB) and scattering function baseline calculation (Python). DOI: 10.1107/S1600576726006187/pag5004sup3.zip

This supplemental material discusses the correlation between the scale and extinction factors. DOI: 10.1107/S1600576726006187/pag5004sup4.pdf

CCDC reference: 2562362

## Figures and Tables

**Figure 10:**
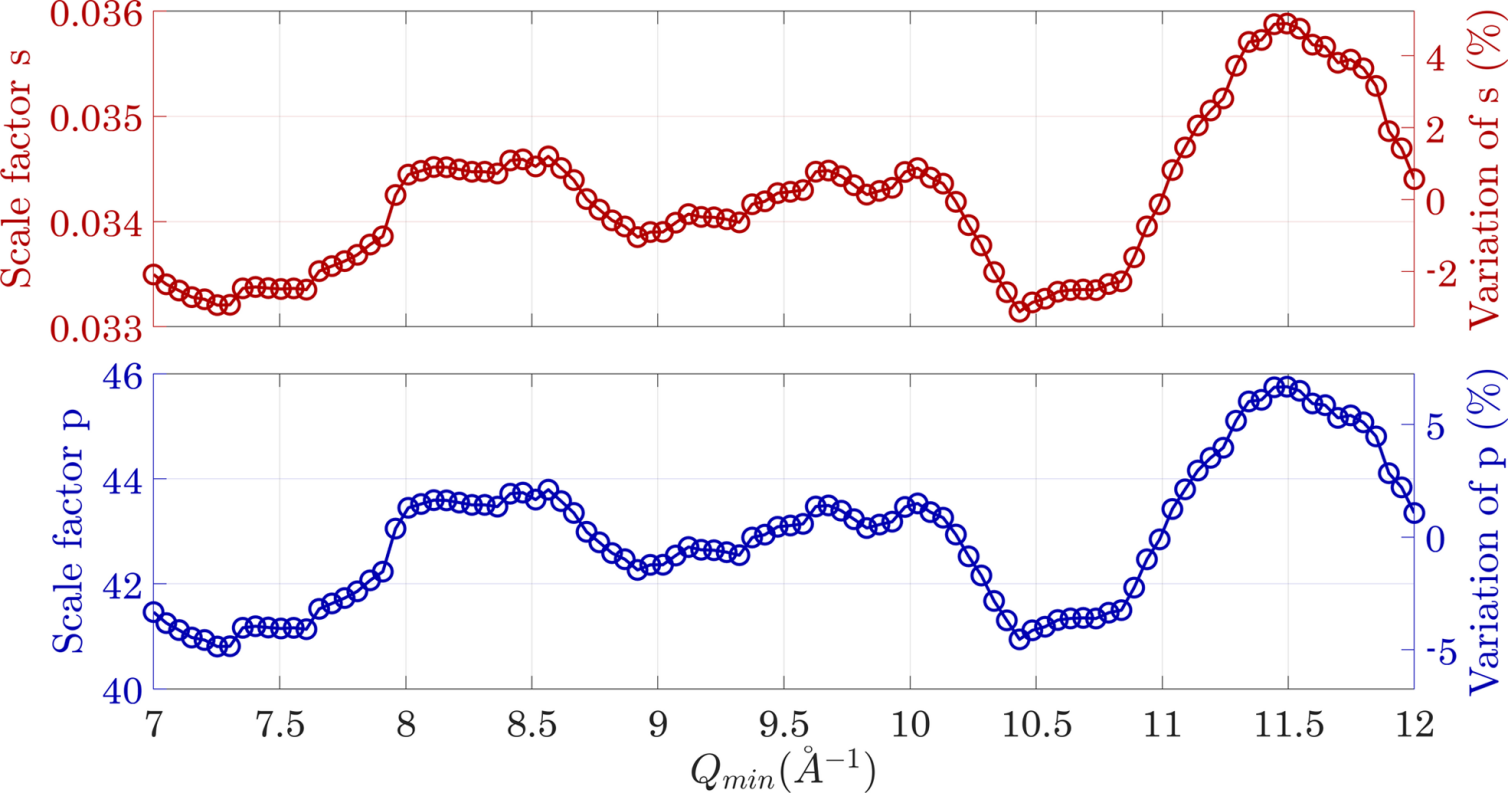
Dependences of the scaling coefficients *s* and *p* on *Q*_min_.

**Table 1:** Comparison of structural refinements for the eight-site and six-site Pb models in PbMg_1/3_Nb_2/3_O_3_ using data from the Bruker D8 Venture diffractometer and the ID28 beamline at ESRF Space group *Pm*3*m*; lattice parameter *a* = 4.0451 (1) Å. In the eight-site model, Pb atoms occupy *x* *x* *x* positions; in the six-site model, they occupy *x* 0 0 positions. Atomic displacement parameters *U*_*ij*_ are given in Å^2^; for Nb and Mg, *U* is isotropic and constrained to be equal for both species. Values in parentheses represent one standard deviation in the last significant digit, as estimated by *ShelXle*. For the ID28 refinement, the Nb site fraction was fixed.

Parameter	*x*_Pb_	Nb fraction	*U*_11_(Pb)	*U*_23_(Pb)	*U*(Nb)	*U*_11_(O)	*U*_33_(O)
Eight-site							
Bruker	0.0391 (3)	0.652 (14)	0.0228 (9)	−0.0059 (3)	0.0085 (5)	0.011 (2)	0.026 (2)
ID28	0.0391 (4)	0.667	0.0237 (8)	−0.0064 (6)	0.0102 (5)	0.011 (2)	0.028 (2)

Six-site							
Bruker	0.0684 (4)	0.652 (14)	0.011 (1)	0.027 (1)	0.0085 (5)	0.010 (2)	0.027 (2)
ID28	0.0694 (8)	0.667	0.013 (1)	0.030 (1)	0.0114 (7)	0.008 (3)	0.030 (4)

## Footnotes

1 Certain equipment, instruments, software or materials, commercial or noncommercial, are identified in this paper in order to specify the experimental procedure adequately. Such identification is not intended to imply recommendation or endorsement of any product or service by NIST, nor is it intended to imply that the materials or equipment identified are necessarily the best available for the purpose.

## Appendix A Conversion between the instrumental and scattering vector coordinates

This appendix summarizes the relationship between the components of the scattering vector **Q** = (*Q*_*x*_, *Q*_*y*_, *Q*_*z*_) and the instrumental coordinates *x*_d_, *y*_d_, φ, ω, χ, where

 *x*_d_, *y*_d_ are detector-plane coordinates of a given detector pixel,

 φ is the crystal rotation angles around the corresponding Eulerian cradle axis,

 ω and χ are the other two Eulerian angles (typically held constant during the acquisition).

The scattering vector **Q** is defined in the laboratory coordinate system corresponding to the diffractometer configuration where all Eulerian angles (ω, χ, φ) are set to zero.

We use the standard definition of the scattering vector:

Here

 λ is the X-ray wavelength,

 **e**_1_ is the unit vector pointing from the crystal to the X-ray source,

 **d** is the unit vector pointing from the crystal to the detector pixel.

The vector **d** = **D**_*xy*_/*D*_*xy*_ is given by the normalized detector-pixel position vector **D**_*xy*_:

where

 *D*_0_ is the distance from the sample to the detector plane,

 **d**_0_ is the unit vector that is normal to the detector plane,

 **x**_d_ and **y**_d_ are the unit vectors defining the detector axes.

Equations (20) and (21) allow us to calculate the coordinates of the vector . To obtain the corresponding scattering vector in the unrotated laboratory frame, we apply the inverse of the rotation matrix [*M*_ωχ_(φ)]:

The total rotation matrix [*M*_ωχ_(φ)], as defined by *e.g.* Gorfman *et al.* (2021 ▸), is the product of three elementary rotations:

All the coordinate axes are summarized in Fig. 2 ▸.

## Appendix B Relationship between measured and theoretical intensities

This appendix summarizes key elements of the kinematical X-ray scattering theory that relate the measured intensity *J*_lab_(*x*_d_, *y*_d_, φ) to the calculated coherent scattering intensity *I*_coh_(**Q**).

We begin by defining the measured quantity *J*_lab_(*x*_d_, *y*_d_, φ), which represents the number of photons accumulated at the detector pixel (*x*_d_, *y*_d_) during the continuous rotation of the crystal over an angular range [φ, φ + Δφ] and exposure time τ:

Here *J*_flux_(*x*_d_, *y*_d_, φ) is the instantaneous photon flux (in photons s^−1^) arriving at the pixel during the rotation. This flux can be related to the local flux density *J*_fd_(*x*, *y*, φ) (in photons s^−1^ mm^−2^) via integration over the pixel area *S*, accounting for the oblique incidence angle η (the angle between the scattered ray and the detector normal):

According to the kinematical theory of X-ray scattering (Warren, 1990 ▸; Guinier, 1994 ▸; Als-Nielsen & McMorrow, 2011 ▸), the local flux density is given by

Here

  is the incident beam flux density (measured in photons s^−1^ mm^−2^). We assume that it is proportional to the reading of the primary beam monitor *B* with  being the proportionality factor that remains constant during the experiment.

 *r*_e_ is the classical electron radius.

 *D* is the sample-to-pixel distance.

 *P*(*x*, *y*) = sin^2^ψ(*x*, *y*) is the polarization factor.

 *A*′(*x*, *y*, φ) is the scattering amplitude corresponding to detector position (*x*, *y*) at rotation angle φ.

To simplify notation, we define the sample-to-detector distance as *D*_0_ = *D* cos η and re-write (24) as

Next, we use the functional dependence between the instrumental (*x*, *y*, φ) and the scattering vector (*Q*_*x*_, *Q*_*y*_, *Q*_*z*_) in the unrotated reference frame as described in Appendix *A*[App appa] as **Q**(*x*, *y*, φ). Substituting *A*′(*x*, *y*, φ) = *A*(*Q*_*x*_, *Q*_*y*_, *Q*_*z*_), equation (27) becomes

We now introduce the generalized Lorentz factor as the inverse Jacobian determinant:

which allows a change of variables:  =  Substituting this into equation (28), we obtain

Although, both the Lorentz factor *L* and polarization factor *P* vary across the detector, they can be treated as constant over the small reciprocal-space volume δ covered by the single 3D pixel. This allows an alternative expression for the Lorentz factor in terms of the volume ratios:

so that equation (30) is transformed into

Applying the multiplicative correction factor as defined in (3) and (4) of the main text,

we obtain the corrected scattering intensity as

Here, *R*_0_ accumulates the factors that remain constant during the experiment:

The reconstruction procedure was introduced in the main text: it involves converting the corrected intensity  into *I*_cryst_(*H*, *K*, *L*) as

which means

Here, the averaging is performed over the reciprocal-space voxel corresponding to the coordinates *HKL*.

Finally, we recall the standard kinematical theory expression for the scattering amplitude:

This is directly connected to the definition of the coherent scattering intensity (2)

which leads to equation (7) in the main text:

## Appendix C Compton scattering

The intensity of Compton scattering is calculated as

where *K*(*Q*) is known as the Klein–Nishina factor:

Here, *E* and *E*′ are the energies of the incident and incoherently scattered photons, respectively. *E*′ is determined through

and *E*_e_ = 0.511 MeV is the rest energy of a free electron.

The incoherent scattering function *S*_inc_(*Q*) is evaluated as an incoherent sum of contributions from all the atoms in the unit cell

where the sum runs over *N*_u_ atoms in the unit cell,  denotes the partial occupancy of the μth atom and *s*_inc,μ_(*Q*) is the tabulated incoherent scattering factor for the corresponding chemical element (Balyuzi, 1975 ▸).

Fig. 9 ▸ shows the relative contribution of the Compton scattering intensity, defined as *I*_compt,r_(*Q*) = *pI*_compt_(*Q*)/*I*(*Q*) with the scale factor *p* determined as described in Section 3.3.

## Appendix D Dependence of the scale factors on *Q*_min_ and estimation of their uncertainties

Fig. 10 ▸ illustrates how the choice of *Q*_min_ affects the resulting scaling coefficients. The corresponding variations reflect both the statistical uncertainty in the *I*(*Q*) values and the systematic uncertainty associated with the scaling procedure.
