# Supplementary material for: Measurements and scaling of X-ray total scattering from single crystals
Source: J Appl Crystallogr. 2026 Jul 24;59(Pt 4):1224–37. doi: 10.1107/S1600576726006187 (PMC13440055; doi:10.1107/S1600576726006187)
Supplement: Supplementary file 1 [file j-59-01224-sup4.pdf]

JOURNAL OF APPLIED CRYSTALLOGRAPHY

Volume 59 (2026)

Supporting information for article:

Measurements and scaling of X-ray total scattering from single crystals

S. Gorfman, M. Eremenko, V. Krayzman, A. Bosak, P. Y. Zavalij and Igor Levin

Dependence of the results of Crystallographic Refinement on the Extinction Parameter

This supplemental section examines how the results of the crystallographic refinement depend on the value of extinction parameter, when such parameter is fixed to some pre-set value. In the original refinement performed using ShelXle, the extinction coefficient x was treated as an independent fitting parameter within the empirical extinction correction expression:

$$\left|F_{corr}^{calc}\right|^2 = s_F\left(1 + 0.001\,x\,\frac{\left|F^{calc}\right|^2\lambda^3}{\sin 2\theta}\right)^{-\frac{1}{2}}\left|F^{calc}\right|^2$$

As discussed in the main text, a strong correlation (0.82) was observed between the refined extinction coefficient x and the scale factor s_F. To further investigate this correlation, we repeated crystallographic refinements using 8-site structural model while systematically varying x over a series of fixed preset values. Because ShelXle does not support refinements with a fixed extinction parameter, these calculations were carried out using a custom MATLAB-based refinement routine.

Figure S1 presents the dependence of the reliability factor $R1$ and the refined value of s_F on the preset extinction coefficient. The result shows that R1 changes only insignificantly over the broad values of x, which explains the strong correlation discussed above. The results demonstrate that the expected value of $s_F = 0.2$ can be reproduced when the extinction coefficient is fixed at x=0.1. However, this constraint leads to a noticeable increase in the reliability factor, with R1 increasing to approximately 0.09 compared to the value of approximately 0.045 obtained in the unconstrained refinement.

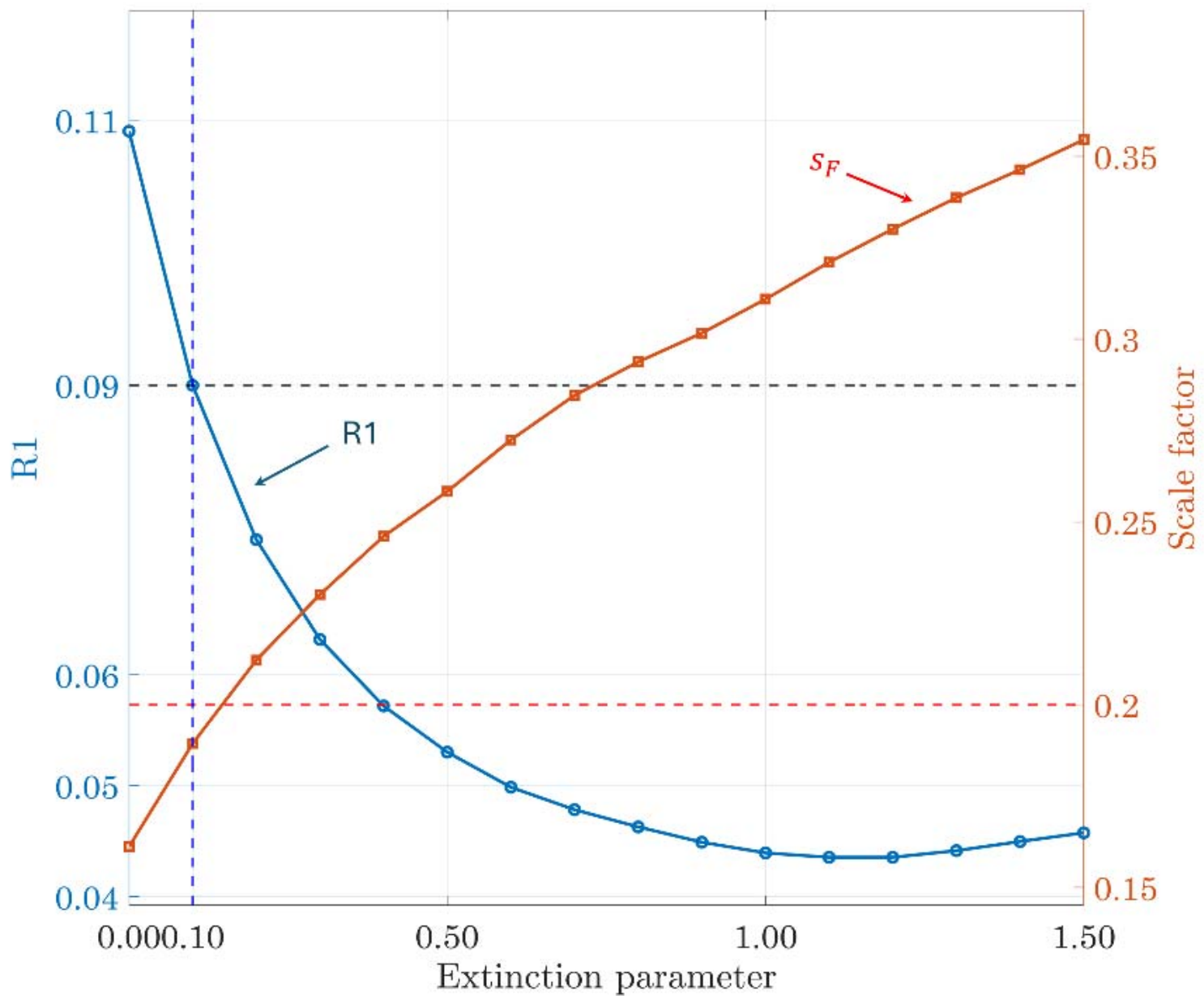

Figure S1. Dependence of the reliability factor R1 and the refined scale factor s_F on the preset value of the extinction coefficient x.
